# Supplementary material for: End Sequence Analysis Toolkit (ESAT) expands the extractable information from single-cell RNA-seq data
Source: Genome Res. 2016 Oct;26(10):1397–410. doi: 10.1101/gr.207902.116 (PMC5052061; doi:10.1101/gr.207902.116)
Supplement: Supplemental Material [file supp_gr.207902.116_Supp_Materials.tar.gz › ESAT-esat_v0.1/doc/html/classjaligner_1_1matrix_1_1_matrix_generator.html]

ESAT: jaligner.matrix.MatrixGenerator Class Reference


ESAT


- Main Page
- Related Pages
- Packages
- Classes
- Files

- Class List
- Class Index
- Class Hierarchy
- Class Members

All Classes Namespaces Files Functions Variables Enumerator Pages

- jaligner
- matrix
- MatrixGenerator

Static Public Member Functions |
List of all members

jaligner.matrix.MatrixGenerator Class Reference

|  |  |
| --- | --- |
| Static Public Member Functions | |
| static Matrix | generate (float match, float mismatch) |
|  | |

## Detailed Description

A utility class to generate a scoring matrix from a fixed match and mismatch scoring scheme.

Author
:   Ahmed Moustafa (ahmed.nosp@m.@use.nosp@m.rs.sf.nosp@m..net)

## Member Function Documentation

|  |  |  |  |
| --- | --- | --- | --- |
| static Matrix jaligner.matrix.MatrixGenerator.generate | ( | float | *match*, |
|  |  | float | *mismatch* |
|  | ) |  |  |

static

Returns scoring matrix from a fixed match/mismatch scoring scheme.

Parameters
:   |  |  |
    | --- | --- |
    | match | match score |
    | mismatch | mistmatch score |

Returns
:   scoring matrix

---

The documentation for this class was generated from the following file:

- java/jaligner/matrix/MatrixGenerator.java


---

Generated on Fri Aug 15 2014 15:11:06 for ESAT by  

 1.8.7
